# An Australian collector's authority file, 1973–2020

**DOI:** 10.3897/BDJ.9.e70463

**Published:** 2021-07-28

**Authors:** Robert Evan Mesibov

**Affiliations:** 1 No affiliation for this profile, West Ulverstone, Tasmania, Australia No affiliation for this profile West Ulverstone, Tasmania Australia

**Keywords:** Australia, Australian Capital Territory, New South Wales, Queensland, South Australia, Tasmania, Victoria, collector, collecting event

## Abstract

**Background:**

Biodiversity databases contain omissions and errors, including those resulting from data entry mistakes and from the use of outdated or incorrect data sources. Some of these omissions and errors can be minimised by the use of authority files, such as expert-compiled taxonomic name databases. However, there are few publicly available authority files for collecting events, and the "where", "when" and "by whom" of specimen data are typically entered into biodiversity databases separately and directly, item by item from specimen labels.

**New information:**

Here I describe a publicly available compilation of 3829 of my own collecting events over a 48-year period in Australia. Each record contains a unique combination of date, georeferenced location and location notes.

## Introduction

Most of the authority files used in biodiversity informatics are databases of taxonomic names, such as those made available by WoRMS and the Catalogue of Life. An index of botanists has been developed by Harvard University Herbaria and Libraries that includes both taxonomists and collectors, and Groom et al. (2020) have argued for online resources that would include all specimen collectors.

Publicly available authority files for collecting events are much rarer, although these do exist within individual institutions. For example, the Queensland Museum Entomology Collection can provide to interested researchers an events table with locality name, georeference, upper and lower elevation, starting and finishing date, collector name(s) and collecting method. The table covers thousands of collecting events, mainly in Queensland, dating back to the early years of the 20th century.

In the past, tables of collecting events were familiar items in the reports of major scientific expeditions. Documentation of the HMS Challenger voyage in the 1870s includes a table (Anonymous 1885) with date, location, collecting method and sea and bottom conditions for more than 500 sampling locations. The 1905 Michaelsen-Hartmeyer expedition to Western Australia reported its results with a list of numbered collection stations and dates, and a map with the numbered stations clearly marked (Michaelsen and Hartmeyer 1907).

In contrast, the BushBlitz program in Australia has not required participants to report collecting event data from the 40 Bush Blitz expeditions undertaken since 2010. Each expedition produces a report with a narrative of field work and a summary of results, but individual collecting events might only be documented as uniform dots on maps, e.g. "Appendix B: Collecting sites" (Bush Blitz 2020). Records of collecting events are the responsibility of individual Bush Blitz participants, and specimen occurrence records are only expected to appear, eventually, in the Atlas of Living Australia (ALA), or in scientific publications. A complete, correct and consistently formatted authority file of collecting events for each Bush Blitz expedition would be useful to participants, natural history collections and ALA.

Less formal but still valuable sources of collecting event data are digitised field books, such as those made available by The Field Book Project of the Smithsonian Institution Archives. Field book data typically require interpretation and reformatting, but they are excellent raw material for authority files.

As with other look-up tables, an authority file for collecting events allows users to avoid database entry errors and to correct errors in data items already in databases. Users may also find information in the authority file that was unintentionally omitted from database entries based on specimen labels, or deliberately excluded in compliance with institutional databasing rules.

I compiled the authority file presented here in Darwin Core format after noticing that museum data in ALA included occurrence records attributed to me as collector with wrong dates and georeferences. The errors presumably arose when museum staff or volunteers entered data incorrectly from labels or data files I had provided. I also found "false positive" records in which I was erroneously listed as a collector. I contacted the relevant institutions asking that these errors be corrected, but there are undoubtedly other such mistakes and omissions in ALA and in museum databases.

I hope the dataset offered here will be of value in future for checking and completing museum database entries, and for avoiding the need for individual institutions to independently georeference my collecting localities. How authority-file information can best be incorporated into collection databases is a matter for institutional data mangers to decide. Users are welcome to contact me for clarifications and for additional information about particular collecting events, and minor updates to the authority file (additional events) may appear in future as new versions in Zenodo (https://doi.org/10.5281/zenodo.4990402).

## General description

### Purpose

Beginning in 1975 I kept written records of my plant and invertebrate collecting events in Australia. In the mid-1990s the accumulated written records, backdated to 1973, were entered in digital files, together with more recent digital records. The data items in the records were primarily the locality names or descriptors, the georeferences and the dates, i.e. the "where" and "when" of each event. For many events I also recorded landform and vegetation details in field books and diaries, but almost never the "what" of the event, namely the identity and number of specimens collected. My digital "where" and "when" records were maintained, corrected and updated until I stopped collecting in 2020.

From 1987 to 1997 I was employed as a contract collector on 27 invertebrate sampling projects (noted in the authority file), each of which generated a contractor's report. These reports are mainly unpublished "gray literature" held in institution and government agency libraries. Most of the reports contain more information about individual collecting sites than is included in the authority file.

The authority file is not quite complete. I sometimes collected particular taxa on request for specialists in Australia and elsewhere, and although I always passed on collection details with the the specimens, I did not always enter those details in my digital records. These missing events are mainly from the 1980s and 1990s; if I learn more about these events I will add them to the latest version of the authority file in Zenodo (https://doi.org/10.5281/zenodo.4990402).

There are also a few events in the authority file which do not have any associated specimens. These events were failed searches for target species in millipede mapping studies. Unfortunately I cannot yet reliably identify which of these failed searches were true blanks, with nothing collected, and which were "target blanks", with non-target species collected and sent to a specialist or museum.

For convenience of use, all fieldnames in the authority file are Darwin Core terms. The only non-standard usage is in samplingProtocol, which includes data items with sampling plot size.

## Geographic coverage

### Description

The dataset contains three events from the Australian Capital Territory, 122 from New South Wales, 17 from Queensland, 41 from South Australia, 3332 from Tasmania and 314 from Victoria. There are no events from the Northern Territory, Western Australia or the Australian island territories. All locations are terrestrial habitats.

### Coordinates

-43.5339 and -17.2539 Latitude; 136.8521 and 153.5506 Longitude.

## Temporal coverage

### Notes

1973-07-07 to 2020-06-02

## Usage licence

### Usage licence

Creative Commons Public Domain Waiver (CC-Zero)

## Data resources

### Data package title

Australian collecting events for Robert Mesibov, 1973-2020

### Resource link

https://doi.org/10.5281/zenodo.4990402

### Number of data sets

1

### Data set 1.

#### Data set name

Australian collecting events for Robert Mesibov, 1973–2020

#### Number of columns

27

#### Description

Dates, georeferenced locations and associated data for 3829 unique collecting events in Australia from 1973 to 2020 with Robert Mesibov as sole, primary or associated collector. The full dataset is provided as a supplementary file (Suppl. material 1) and as a versionable resource in Zenodo.

**Data set 1.** 

Column label	Column description
eventID	Unique identifying code for each event: REM-event-0001 to REM-event-3829 in the current version with 3829 events
country	Name of the country ("Australia")
countryCode	ISO 3166-1-alpha-2 country code ("AU")
stateProvince	Full name of the Australian state or territory
verbatimLocality	Brief text description of the location
decimalLatitude	Latitude of the location's center, in decimal degrees to 4 decimal places
decimalLongitude	Longitude of the location's center, in decimal degrees to 4 decimal places
coordinateUncertaintyInMeters	Estimate of the radius of a circle around the specified decimalLatitude and decimalLongitude containing the whole of the location
geodeticDatum	Datum for decimalLatitude and decimalLongitude entries ("WGS84")
coordinatePrecision	Precision of the coordinates in decimalLatitude and decimalLongitude ("0.0001")
verbatimCoordinates	Coordinates originally recorded for the location
verbatimSRS	Datum for the original coordinates
verbatimCoordinateSystem	Format of the original coordinates
georeferenceSources	Device (GPS), institution records, maps and online resources used to georeference the location
georeferencedBy	Person who georeferenced the location, or confirmed or corrected another georeferencer's work ("Mesibov, Robert")
georeferenceRemarks	Comments on georeferencing of particular locations
minimumElevationInMeters	Lower elevation above sea level of the location
maximumElevationInMeters	Upper elevation above sea level of the location
eventDate	ISO-8601 date or interval date for the collecting event (YYYY-MM-DD or YYYY-MM-DD/YYYY-MM-DD)
year	Year of the collecting event (YYYY)
month	Month of the last date in the collecting event (MM)
day	Day of the last date in the collecting event (DD)
recordedBy	Collector names in full for the event, as "last name, first name"
eventRemarks	Comments on the event, e.g. the name of the project which sponsored the collection
locationRemarks	Comments on the location
samplingProtocol	Description of sampling during the event
fieldNumber	Codes assigned to samples from particular events, 1975–1986 (not event codes), pipe-separated

## Additional information

**Location text.** The verbatimLocality entries are the location text strings I used in my field notes and in most of my publications. The text strings may differ in detail from those on hand-written or printed specimen labels I prepared.

**Spatial data.** From the 1970s through the 2000s I used paper maps to estimate locations, and beginning in 2001 I also used a handheld GPS unit and online digital maps. In all cases I later checked the estimated locations using better spatial data resources, e.g. Google Earth or the online mappers provided by Australian state governments. These checks sometimes resulted in small changes in georeferences or spatial uncertainties. For practical reasons I did not provide updated spatial data to the museums holding original specimen labels, and label locations may therefore differ slightly from locations in the authority file. The originally recorded format for spatial data also changed during the dataset period, from UTM coordinates to latitude/longitude, and from the AGD66 datum to GDA94 (equivalent to WGS84 during the sampling period).

**Spatial uncertainty.** The minimum value in the coordinateUncertaintyInMeters field is 25 (apart from eight locations measured along a transect), i.e. the collecting site is within a circle with diameter 50 m centered on the indicated coordinates. This minimum is meant to cover both the area searched for specimens and the GPS uncertainty, which can be considerable in dense forest locations in hilly terrain in Tasmania, where I did most of my collecting. Larger uncertainty values in this field mean that I collected over a larger area or that the scale of the map used to locate collecting sites did not allow for more exact georeferencing.

**Collector identifiers.** Many of my 60 co-collectors were non-scientists, or scientists without publicly listed identifiers. Below I list the nine currently available ORCID identifiers.

Decker, Peter: https://orcid.org/0000-0003-0345-7738

Edgecombe, Gregory: https://orcid.org/0000-0002-9591-8011

Hill, Lionel: https://orcid.org/0000-0002-8686-4015

Johanson, Zerina: https://orcid.org/0000-0002-8444-6776

Korsós, Zoltán: https://orcid.org/0000-0003-1545-5086

Mesibov, Robert: https://orcid.org/0000-0003-3466-5038

Purcell, Michaela: https://orcid.org/0000-0003-0507-2997

Reid, Amanda: https://orcid.org/0000-0001-5765-1363

Rowell, David: https://orcid.org/0000-0002-2077-9774

## Supplementary Material

43678734-5E4A-5D2B-A319-03C91CC528C7 10.3897/BDJ.9.e70463.suppl1 Supplementary material 1 Collecting events Data type Data table Brief description Dates, georeferenced locations and associated data for 3829 unique collecting events in Australia from 1973 to 2020 with Robert Mesibov as sole, primary or associated collector. File: oo_570161.txt https://binary.pensoft.net/file/570161 Robert Mesibov
